# Silk ProteinsEnriched Nanocomposite Hydrogels Based on Modified MMT Clay and Poly(2-hydroxyethyl methacrylate-co-2-acrylamido-2-methylpropane Sulfonic Acid) Display Favorable Properties for Soft Tissue Engineering

**DOI:** 10.3390/nano12030503

**Published:** 2022-01-31

**Authors:** Mirela Violeta Șerban, Simona-Rebeca Nazarie (Ignat), Sorina Dinescu, Ionuț-Cristian Radu, Cătălin Zaharia, Elena-Alexandra Istrătoiu, Eugenia Tănasă, Hildegard Herman, Sami Gharbia, Cornel Baltă, Anca Hermenean, Marieta Costache

**Affiliations:** 1 Department of Biochemistry and Molecular Biology, University of Bucharest, 050663 Bucharest, Romania; mirela.serban@unibuc.ro (M.V.Ș.); simona.ignat@unibuc.ro (S.-R.N.); marieta.costache@bio.unibuc.ro (M.C.); 2 The Research Institute of the University of Bucharest (ICUB), University of Bucharest, 050663 Bucharest, Romania; 3 Advanced Polymer Materials Group, University Politehnica of Bucharest, 011061 Bucharest, Romania; radu.ionucristian@gmail.com (I.-C.R.); zaharia.catalin@gmail.com (C.Z.); alexandra.rata20@gmail.com (E.-A.I.); eugenia.vasile27@gmail.com (E.T.); 4 “Aurel Ardelean” Institute of Life Sciences, ”Vasile Goldiș” Western University of Arad, 310025 Arad, Romania; hildegard.i.herman@gmail.com (H.H.); samithgh2@gmail.com (S.G.); baltacornel@gmail.com (C.B.)

**Keywords:** modified MMT, nanocomposite hydrogels, sericin, fibroin, adipogenesis

## Abstract

Due to their remarkable structures and properties, three-dimensional hydrogels and nanostructured clay particles have been extensively studied and have shown a high potential for tissue engineering as solutions for tissue defects. In this study, four types of 2-hydroxyethyl methacrylate/2-acrylamido-2-methylpropane sulfonic acid/montmorillonite (HEMA/AMPSA/MMT) hydrogels enriched with sericin, and fibroin were prepared and studied in the context of regenerative medicine for soft tissue regenerative medicine. Our aim was to obtain crosslinked hydrogel structures using modified montmorillonite clay as a crosslinking agent. In order to improve the in vitro and in vivo biocompatibility, silk proteins were further incorporated within the hydrogel matrix. Fourier transform infrared spectroscopy with attenuated total reflectance (FTIR-ATR) were performed to prove the chemical structures of the modified MMT and nanocomposite hydrogels. Swelling and rheological measurements showed the good elastic behavior of the hydrogels due to this unique network structure in which modified MMT acts as a crosslinking agent. Hydrogel biocompatibility was assessed by MTT, LDH and LIVE/DEAD assays. The hydrogels were evaluated for their potential to support adipogenesis in vitro and human stem cells isolated from adipose tissue were seeded in them and induced to differentiate. The progress was assessed by evaluation of expression of adipogenic markers (*ppar-γ2*, *perilipin*) evaluated by qPCR. The potential of the materials to support tissue regeneration was further evaluated on animal models in vivo. All materials proved to be biocompatible, with better results on the 95% HEMA 5% AMPSA enriched with sericin and fibroin material. This composition promoted a better development of adipogenesis compared to the other compositions studied, due the addition of sericin and fibroin. The results were confirmed in vivo as well, with a better progress of soft tissue regeneration after implantation in mice. Therefore, hydrogel 95% HEMA 5% AMPSA enriched with sericin as well as fibroin showed the best results that recommend it for future soft tissue engineering application.

## 1. Introduction

Over the years, the fields of tissue engineering and regenerative medicine have evolved constantly in order to develop better tissue-like constructs that can repair an injury or replace an affected organ. There is a growing need for various soft tissue defects (skin injuries, breast reconstruction etc.) solutions that can be addressed by soft tissue engineering [1]. It is important to create the perfect combination of materials, type of cells and biochemical and physical factors that match the specific needs of a patient.

Materials should mimic the targeted tissue in order to integrate better in the implanted environment. Moreover, they should support cellular adhesion, proliferation and differentiation for proper tissue regeneration [2,3]. Hydrogels are 3D networks with the ability to absorb large amounts of water. Hydrogels can be made with natural or synthetic polymers and represent a great candidate for soft tissue engineering due to their biomimetic properties and multifunctionalities [4]. Many polymers have been investigated for their potential, but the best results were obtained when using a combination of more than two types of polymers to benefit their advantages simultaneously. Natural or synthetic materials could be used such as chitosan, alginate, collagen, fibrin, hyaluronic acid or polyacrylic acid, polyacrylamide, poly(*N*-isopropylacrylamide), both carrying advantages for medical use.

2-hydroxylethyl methacrylate (HEMA) was used in different hydrogel combinations with other materials, and its biocompatibility and potential biomedical use was confirmed [5]. In addition, poly(2-hydroxyethyl methacrylate) (PHEMA) was shown to have potential in soft tissue engineering [2,6]. Furthermore, poly(2-hydroxyethyl methacrylate-co-2-acrylamido-2-methylpropane sulfonic acid) (p(HEMA-AMPSA)) hydrogel developed by Hu et al. [7] promoted cellular adhesion and indicated a potential use for soft tissue engineering.

The addition of clays in hydrogels represents an attractive option that can improve the properties of the final material [8]. Several important developments were performed, which have expanded the use of hydrogel materials. The introduction of crosslinking agents, double network hydrogels, and nanocomposite (clay filled) hydrogels have significantly improved the mechanical properties of hydrogels [9]. Nanocomposite clay hydrogels are organic–inorganic hybrids that show superior mechanical properties [9,10,11,12]. Various types of clay materials have been used such as montmorillonite (MMT), bentonite, kaolinite or layered double hydroxides [13,14,15].

Two major proteins from silk, fibroin and sericin, showed great results for tissue engineering applications [16]. Fibroin has impressive mechanical properties and a relatively slow degradation rate [17,18]. It can be added to different materials in order to provide more cell recognition sites and promote cell–scaffold interaction [17]. Sericin was also used in many tissue engineering studies and was shown to enhance the overall properties of the materials by promoting cellular adhesion, cell proliferation and biodegradation [19,20].

Depending on the tissue engineering application, different type of cells could be used. Adipose tissue-derived stem cells (ASCs) are gaining interest due to their potential to differentiate toward multiple types of lineages (osteogenic, adipogenic, chondrogenic, etc.), and they are easily obtained from lipoaspirates [21,22,23,24,25].

The present study aims to evaluate the potential of newly developed materials based on poly(2-hydroxyethyl methacrylate-co-2-acrylamido-2-methylpropane sulfonic acid) with modified clay and enriched with sericin and fibroin for soft tissue engineering applications. We investigated their properties, biocompatibility, and the potential to support adipogenesis both in vitro and in vivo.

## 2. Materials and Methods

The monomers 2-hydroxyethyl methacrylate (HEMA), 2-acrylamido-2-methylpropane sulfonic acid (AMPSA), initiator potassium persulfate (KPS), clay montmorillonite (MMT K10), benzene-1,2,4-tricarboxylic acid 1,2-anhydride (TA) and silk sericin (quality level 200) were purchased from Sigma Aldrich. *Bombyx mori* cocoons were kindly provided by SC Sericarom SA, sodium carbonate (Na_2_CO_3_) and sodium bicarbonate (NaHCO_3_) by Aldrich, sodium dodecyl sulfate was obtained from Merck and lithium bromide (LiBr) was purchased from Honeywell. Silk fibroin was obtained from silkworm cocoons by the degumming method [26]. Briefly, Chopped *Bombyx mori* cocoons were degummed by washing three times in hot aqueous solution of 0.5% (*w*/*v*) Na_2_CO_3_, NaHCO_3_ and sodium dodecyl sulfate, and then rinsed thoroughly with distilled water to extract the sericin protein and other components. The purified silk fibroin was dried at 40 °C and atmospheric pressure. The extracted silk fibroin was then dissolved in a 9.3 M LiBr solution at 60 °C for 6 h, yielding an approximately 2% (*w*/*v*) solution. This solution was dialyzed in distilled water with a dialysis tubing cellulose membrane (molecular weight cutoff, MWCO, 12 kDa) for 3 days with frequent water changes. The final concentration of the silk fibroin aqueous solution was 2 wt.%, which was determined by weighing the remaining solid after drying.

### 2.1. Materials Synthesis and Characterization

#### 2.1.1. Synthesis of Modified MMT by Reaction with Benzene-1,2,4-tricarboxylic Acid 1,2-Anhydride (MMT-TA)

First, montmorillonite reacted with benzene-1,2,4-tricarboxylic acid 1,2-anhydride (TA) for tailoring carboxylic groups. The synthesis involved MMT dispersion in acetone by sonication (0.5% *w*/*v*). In order to have a suitable pH for the esterification reaction (pH 3), HCl 0.1M was added to the previously obtained dispersion (10% *v*/*v*). The last synthesis step assumed benzene-1,2,4-tricarboxylic acid 1,2-anhydride (TA) addition (MMT:TA-1:4 *w/w*). The reaction lasted for 48 h at room temperature. The synthesis product was purified by repeated washing with fresh acetone and dried at 60 °C.

#### 2.1.2. MMT-TA Modification by Reaction with 2-Hydroxyetylmetacrylaye (MMT-TA-HEMA)

The MMT-TA product was further modified with 2-hydroxyethyl methacrylate (HEMA) in order to introduce double bonds within the layers surface. Thus, dried MMT-TA was added in 2-hydroxyethyl methacrylate (conc. 0.3% *w*/*v*) and sonicated for 1 h. In parallel, an acetone-HCl 0.1M mixture was prepared (4:1 *v*/*v*). This mixture is added to improve the clay dispersion degree into the reaction media. Furthermore, the mixture reaches an acidic pH value (pH 3–4) for the esterification step. The mixture was added in the MMT-TA/HEMA dispersion. The reaction lasted for 24 h at room temperature. The final product was filter washed several times with acetone. In the final step, the product was dried.

#### 2.1.3. Preparation of the Hydrogels from Modified Clay (MMT-TA-HEMA) and HE-MA/AMPSA Monomers

Nanocomposite hydrogels were obtained by sol-gel free radical polymerization of HEMA and AMPSA in the presence of 1% modified clay (MMT-TA-HEMA). The polymerization reaction was initiated by potassium persulfate (KPS) at 60 °C. No crosslinking agent was used. To increase the biocompatibility, silk fibroin and sericin were added in the reaction mixture (Table 1). Therefore, a silk fibroin aqueous solution (2% *w*/*v*) was prepared according to the well-known procedure [26]. AMPSA monomer was dissolved into fibroin solution followed by KPS and sericin addition. In parallel, the MMT-TA-HEMA water dispersion was obtained by sonication for 1 h. The two mixtures were placed together and injected into a glass mold. The glass mold was placed in an oven at 60 °C for 48 h. The obtained nanocomposite hydrogels were purified with distilled water for several days. The recipes for the hydrogel preparation are shown in Table 1. Samples for biological and morphological investigation were frozen and lyophilized (−80 °C/10^−2^ mbar).

#### 2.1.4. Fourier Transform Infrared Spectroscopy-Attenuated Total Reflectance (FTIR-ATR) Characterization

FTIR-ATR physico-chemical investigation of crude clay, modified clay and corresponding hydrogels was performed using a Bruker Vertex 70 FT-IR spectrophotometer with Attenuated total reflectance (ATR) accessory. FT-IR spectrophotometer used 32 scans and a resolution of 4 cm^−1^ in mid-IR region 600–4000 cm^−1^.

#### 2.1.5. Morphological Investigation by Scanning Electron Microscopy (SEM)

Morphological information including internal structure of the hydrogels was obtained through the scanning electron microscopy (SEM) analysis of the gold-coated composites. The analysis was performed using a QUANTA INSPECT F SEM device equipped with a field emission gun (FEG) with a resolution of 1.2 nm and with an X-ray energy dispersive spectrometer (EDS).

#### 2.1.6. Swelling Behavior and Rheological Properties

*Swelling behavior.* The swelling behavior of the nanocomposite hydrogels was evaluated in saline solution at 37 °C. The weight changes of the samples were recorded at regular time intervals during swelling. The swelling degree of the specimens was calculated according to the following equation:

(1) $$SD=\frac{M_{t}-M_{0}}{M_{0}}\cdot$$

where *M_t_* and *M_0_* denote the weight of the wet hydrogel at a predetermined time and the weight of the dry sample, respectively. The equilibrium swelling degrees (ESD) were measured until the weight of the swollen samples was constant. At least three swelling measurements were performed for each sample and the mean values were reported.

*Rheological properties*. Rheological measurements were performed with a rotational rheometer Kinexus Pro, Malvern Instruments, and a temperature control unit. In oscillating mode, a parallel plate and a geometric measuring system were used, and the gap was set according to the force value. The tests were performed on samples of 20 mm diameter with parallel plate geometry in a frequency range 1 to 10 Hz. The water saturated nanocomposite hydrogels were placed on the rheometer test bench and the rheological factors such as elastic modulus G′ and viscous modulus G″ were evaluated.

### 2.2. In Vitro Tests

#### 2.2.1. Biocompatibility Assessment of the Materials

For the biocompatibility tests, human stem cells derived from adipose tissue (hASCs, ThermoFischer Scientific, Whaltam, MA, USA, R7788115) were cultured in DMEM media (Sigma-Aldrich, Darmstadt, Germany) with 10% fetal bovine serum (FSB, ThermoFischer Scientific, Whaltam, MA, USA) and 1% antibiotic antimycotic solution (Sigma-Aldrich, Darmstadt, Germany) at 37 °C. Materials were sterilized and cells were seeded at 2 × 10^5^ cells/cm^2^. The biocompatibility of the materials was evaluated at T1 (2 days after seeding) and T2 (5 days after seeding). MTT test (Sigma Aldrich, Darmstadt, Germany) assessed the cell viability and proliferation rate in contact with the materials. At T1 and T2, media were removed and a solution of 3-(4,5-Dimethylthiazol-2-yl)-2,5-diphenyltetrazolium bromide 1mg/mL was added for 4 h. Cells metabolically active reduced the compound to formazan crystals that were further solubilized in isopropanol. The absorbance of the solution was measured at 550 nm by spectrophotometry and indicated the level of metabolically active cells from all materials.

The LDH test (Tox7, Sigma-Aldrich, Darmstadt, Germany) indicated the materials cytotoxicity induced on cells, by measuring the levels of lactate dehydrogenase released by dead cells in the media. At T1 and T2, 50 μL cell media were collected in a 96-well plate. Then, 100 μL of kit solution (following kit instructions) was added in each well. The absorbance of the solution resulted was measured at 490 nM and indicated the level of dead cells in contact with the materials.

The Live/Dead test (ThermoScientific, Whaltam, MA, USA) is a qualitative test that allows to visualize the live cells (calcein AM, green) and nuclei of dead cells (ethidium homodimer, red). At T1 and T2, cells seeded in the materials were stained with the kit solution (following kit instructions) and they were visualized by confocal microscopy (Nikon A1/A1R Confocal Laser Microscope System, New York, NY, USA).

#### 2.2.2. Biochemical Analysis

To evaluate the potential of the materials to sustain adipose differentiation, cells were seeded in the materials at 4 × 10^5^ cells/cm^2^. After 24 h, adipogenesis was induced with specific media (StemProAdipogenesis Differentiation Kit, Thermo Fischer Scientific, Waltham, MA, USA). Media were changed every 3 days. The expression of specific adipogenic markers (*ppar-γ*,* perilipin*) was evaluated by qPCR after 10 and 21 days of adipogenic induction. Total RNA was isolated from samples using TRIzol Reagent (Thermo Fisher Scientific, Waltham, MA, USA) following the manufacturer’s instruction. Revers transcription was performed in order to obtain complementary DNA using High-Capacity cDNA Reverse Transcription Kit (Thermo Fisher Scientific, Waltham, MA, USA). For Real Time PCR, the SYBR Select Master Mix (ThermoScientific, Waltham, MA, USA) and the reaction was performed on ViiA 7 Real-Time PCR System (ThermoScientific, Waltham, MA, USA). Primers used were: *ppar-γ* F 5′ TTACACAATGCTGGCCTCCTT 3′,* ppar-γ* R 5′ AGGCTTTCGCAGGCTCTTTAG 3′, *perilipin* F 5′ ATGCTTCCAGAAGACCTACA 3′, *perilipin* R 5′ CAGCTCAGAAGCAATCTTTT 3′, *gapdh* F 5′ GAGTCAACGGGGTCGT 3′, *gapdh* R 5′ TTGATTTTGGATCTCG 3′.

### 2.3. In Vivo Experiment

Adult CD1 mice used in this study were housed in individually vented cages, with ad libitum access to food/water, standard conditions of temperature/relative humidity and a light/dark cycle of 12/12 h. The experimental protocol was previously approved by the Ethics Committee of the Vasile Goldis Western University of Arad (approval no.08/17.02.2020). The mice were randomly assigned to 5 groups (*n* = 10), as follows:

- Group 1 (control group) without any material;
- Group 2 (M1 group) implanted material containing MMT-5 mol/L-H95%-A5% with Sericin and Fibroin;
- Group 3 (M2 group) implanted material containing MMT-5 mol/L-H97%-A3% with Sericin and Fibroin;
- Group 4 (M3 group) implanted material containing MMT-5 mol/L-H95%-A5%;
- Group 5 (M4 group) implanted material containing MMT-5 mol/L-H97%-A3%.

**Figure 1:**
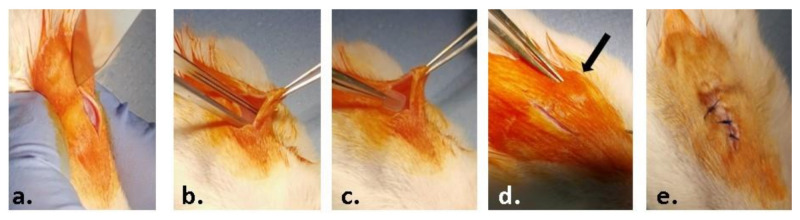
Steps of the surgical procedure: (**a**) preoperative asepsis of the region of interest and incision of the skin on a median dorsal level; (**b**) detachment of the skin from the muscular substrate; (**c**) inserting the test material; (**d**) closing the edges of the wound; (**e**) suturing and post-operative asepsis of the wound.

The material samples (0.5 × 0.5 cm) were implanted into a subcutaneous pocket in the left part of the dorsum of the animals, under anesthesia by intraperitoneal injection of xylazine/ketamine (Figure 1). The animals were euthanized one week and three weeks post-surgery by anesthetic overdose and blood and the implanted materials, together with the surrounding tissues, were explanted and collected for analysis.

#### 2.3.1. Biochemical Analysis

The collected blood samples were centrifuged at 3000 rpm for 10 min and analyzed for C-reactive protein (CRP) levels, using the CRP FL (ChemaDiagnostica, Monsano, Italy) kit and a Mindray BS-120 Chemistry Analyzer (ShenzenMindray Bio-Medical Electronics Co., Ltd., Nanshan, Shenzhen, China).

#### 2.3.2. Histology and Immunohistochemistry

Adipose tissue regeneration was assessed by histological and immunohistochemical analyzes (adipogenic markers).

For the histopathological study, explant samples were fixed in phosphate-buffered formaldehyde solution (4%, pH 7.2, 0.05 M). After dehydration and clarification, samples were embedded in paraffin. After sectioning at 5 µm thickness, sections were stained with hematoxylin and eosin (H&E) and Gomori’ s trichrome kit (Leica Biosystems, 38016SS1, Nussloch, Germany) according to the manufacturer’s protocol.

For the immunohistochemical studies the paraffin-embedded explant tissue sections were previously deparaffinized and rehydrated using a standard technique. After heat-mediated antigen retrieval in citrate buffer (pH 6.5), the sections were incubated overnight at 4 °C with the primary antibody. Rabbit polyclonal anti-tumor necrosis factor (TNF)-α diluted 1:100 (Santa Cruz, CA, USA) was used as a primary antibody. Immuno-reactions were visualized employing a Novocastra Peroxidase/DAB kit (Leica Biosystems, Nussloch, Germany) according to the manufacturers’ instructions.

All the microscopic sections were analyzed with an Olympus BX43 microscope equipped with a digital camera Olympus XC30 and CellSense software.

## 3. Results

### 3.1. Materials Synthesis and Characterization

#### 3.1.1. MMT Modification Pathway

**Figure 2:**
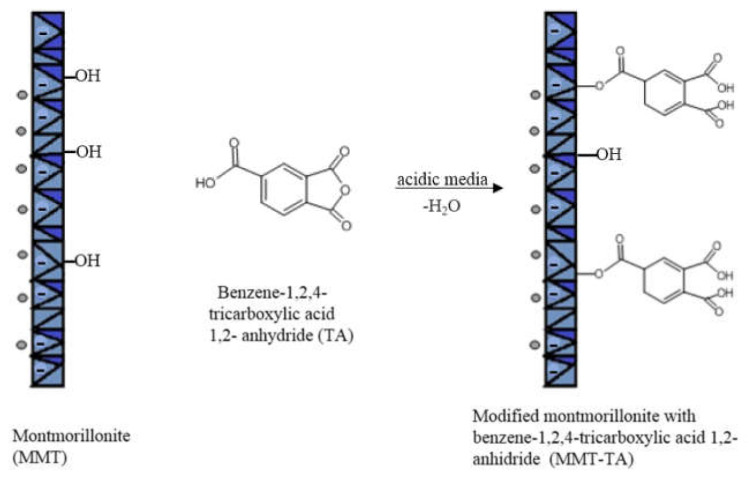
Reaction mechanism of MMT with benzene-1,2,4-tricarboxylic acid 1,2-anhydride.

**Figure 3:**
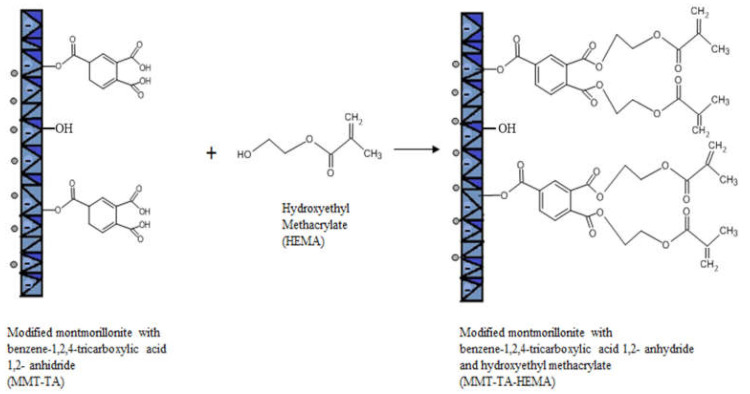
Reaction mechanism of MMT-TA with 2-hydroxyethyl methacrylate.

The modifications of MMT (MMT-TA and MMT-TA-HEMA) are shown in Figure 2 and Figure 3. The first step involved an esterification reaction between hydroxyl groups available within clay layers surface and carboxyl group from benzene-1,2,4-tricarboxylic acid 1,2-anhydride (TA). There are similar reactions exploiting the MMT surface hydroxyl groups for chemical grafting besides prevalent ion–ion interactions [27,28]. Furthermore, the TA anhydride has the capacity for esterification and ring opening reaction in acidic media [29]. The reaction required acid media (pH 3). Furthermore, the synthesis focused on the opening of the anhydride cycle and release the two free carboxyl groups. The second synthesis assumed another esterification reaction between the free carboxyl groups from clay surface with hydroxyl groups from 2-hydroxyethyl methacrylate mono-mer. The second synthesis followed the introduction of double bonds within the clay sur-face. These double bonds are available for the reaction with the free monomers (HEMA and AMPSA) in the polymerization step. HEMA monomer was chosen as a double bond source due to its higher compatibility with the free HEMA monomer.

#### 3.1.2. Physico-Chemical Investigation

##### FTIR-ATR Analysis

**Figure 4:**
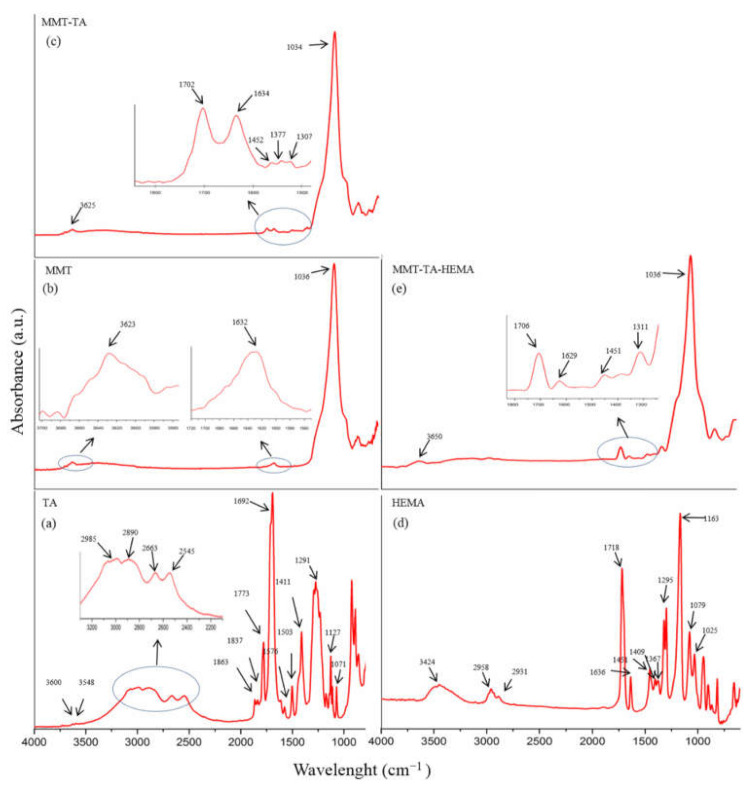
FTIR spectra: benzene-1,2,4-tricarboxylic acid 1,2-anhydride (TA) (**a**); unreacted MMT (**b**); modified MMT with benzene-1,2,4-tricarboxylic acid 1,2-anhydride (MMT-TA) (**c**); 2- hydroxyethyl methacrylate (HEMA) (**d**); MMT-TA modified with HEMA (MMT-TA-HEMA) (**e**).

FTIR-ATR spectra of crude MMT clay, TA and HEMA, and modified MMT-TA and MMT-TA-HEMA are presented in Figure 4. The spectrum of benzene-1,2,4-tricarboxylic acid 1,2-anhydride (TA) (Figure 4a) show several specific peaks in the region 3000–2500 cm^−1^ attributed to C-H stretching vibration. Peaks in the range 1800–1692 cm^−1^ are assigned to carbonyl ring from anhydride. The peaks at 1863 and 1837 cm^−1^ can be assigned to symmetric C=O stretching in anhydride group (weak). Peak at 1863 cm^−1^ can be assigned to asymmetric C=O stretching in anhydride group (strong). Peak at 1692 cm^−1^ can be attributed to symmetric C=O stretching in carboxyl group [30,31].

Figure 4b shows the specific peaks for MMT spectrum. The signals at 3623 and 1632 cm^−1^ are assigned to stretching vibration of O-H group and bending vibration of O-H, respectively. The band at 1036 cm^−1^ corresponds to the stretching vibrations of Si-O groups. Figure 4c illustrates the spectrum of reacted MMT with benzene-1,2,4-tricarboxylic acid 1,2-anhydride (TA). Thus, the MMT-TA spectrum show the following signals: peak at 3625 cm^−1^ is attributed to stretching vibration of O-H groups, peak at 1702 cm^−1^ is attributed to stretching vibration of C=O bonds, peak at 1634 cm^−1^ is assigned to bending vibration of O-H and the last two peaks at 1452 and 1377 cm^−1^ correspond to the bending vibration of C-H. Furthermore, peak at 1307 cm^−1^ is attributed to stretching vibration of -O-C=O ester bonds. Therefore, the peaks at 1452, 1377 and 1307 cm^−1^ confirmed the clay modification by TA carboxyl group [32]. Specific vibrations, such as C-H, on the clay surface can only come from TA anhydride. Peak at 1307 cm^−1^ is the direct contribution of the MMT hydroxyl groups and TA carboxyl groups’ reaction. HEMA spectrum (Figure 4d) shows a peak at 3424 cm^−1^ attributed to the stretching vibration of O-H groups and two peaks at 1377 and 1377 cm^−1^ assigned for C-H stretching vibration. Peaks at 1718 and 1636 cm^−1^ are attributed to carbonyl stretching vibration and carbon double bond, respectively. Peaks in the range 1400–1300 cm^−1^ are assigned to C-H bending vibration. The modified MMT-TA-HEMA spectrum (Figure 4e) reveals the HEMA peaks characteristic for low shifting: 1706 and 1639 cm^−1^ (carbonyl and double bond vibrations). These results can be explained by the presence of HEMA monomer on the clay layer surface. The chemical bonding was revealed by the 1310 cm^−1^ peak responsible for ester groups.

**Figure 5:**
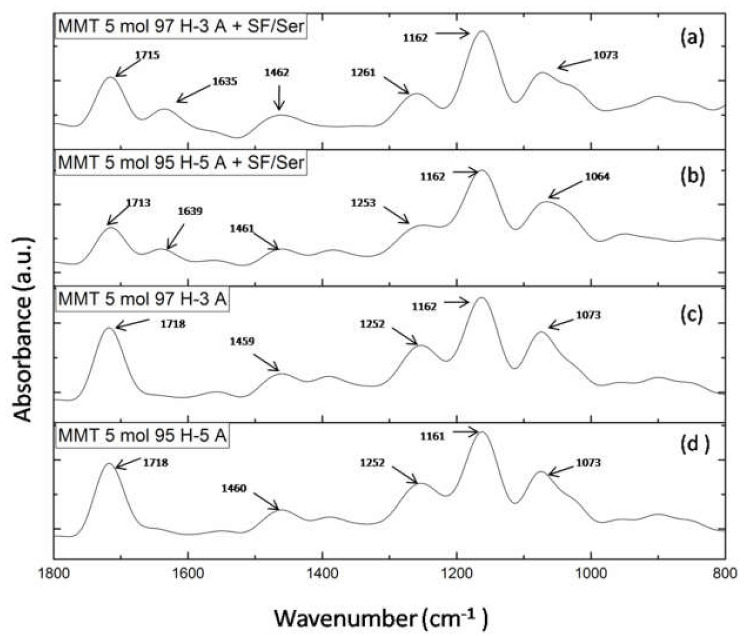
FTIR spectra of: (**a**) MMT-5 mol/L-H97% -A3% with Sericin and Fibroin; (**b**) MMT-5 mol/L-H95%-A5% with Sericin and Fibroin; (**c**) MMT-5 mol/L-H97% -A3%; (**d**) MMT-5 mol/L-H95% -A5%.

Figure 5 presents FTIR spectra for hydrogels with and without proteins for each monomer ratio. FTIR spectra of hydrogels with proteins (Figure 5a,b) reveal the main peaks specific for silk fibroin and sericin. Therefore, the peak at 1715/1713 cm^−1^ is specific for amide I in proteins (C=O) and for carbonyl groups in HEMA and AMPSA units; peak at 1635/1639 cm^−1^ is specific for amide II in proteins (N-H bending and C-N stretching); peak at 1462/1461 cm^−1^ is attributed to methyl/methylene bending in proteins and synthetic units; peak at 1261/1253 cm^−1^ is attributed to amide III in proteins (C-N stretching and N-H bending) and for C-N bond in synthetic units [26,33,34,35]. To summarize, the presence of the two proteins is well highlighted in the spectra even though their concentration was significantly low. FTIR spectra of hydrogel without proteins (Figure 5a,b) lack the peak at 1635 cm^−1^ which is attributed to amide II in proteins. The other peaks are common in the spectra of hydrogel without proteins.

#### 3.1.3. SEM Morphological Characterization

**Figure 6:**
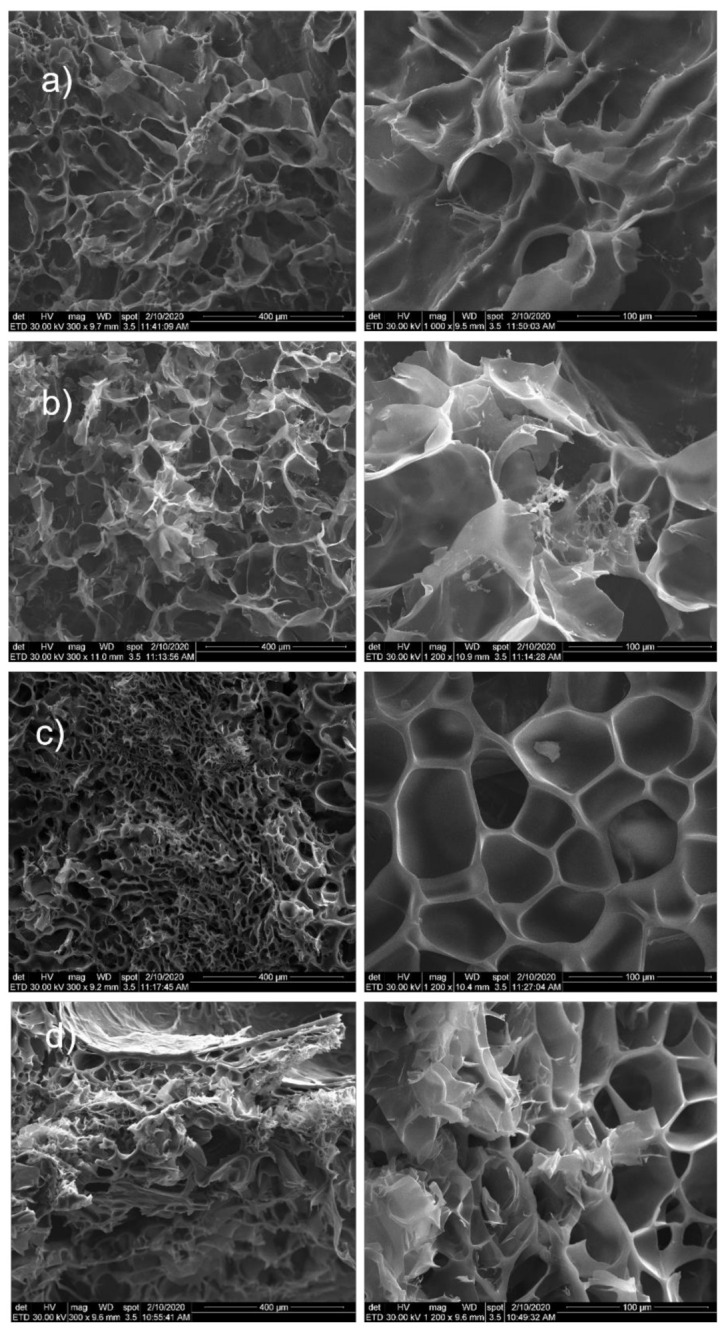
SEM images for: (**a**) MMT-5 mol/L-H95%-A5%; (**b**) MMT-5 mol/L-H97%-A3%; (**c**) MMT-5 mol/L-H95%-A5% with Sericin and Fibroin; (**d**) MMT-5 mol/L-H97%-A3% with Sericin and Fibroin.

Figure 6 shows the morphological images for nanocomposite hydrogels with 5 mol/L monomer molar concentration. Furthermore, samples with 5 mol/L monomer concentration were prepared with different monomer ratio: HEMA97%-AMPSA3% and HEMA95%-AMPSA5%.

All samples showed a porous structure with interconnected and open pores. There are few differences between samples with the two different monomer ratios. Significant modification appeared between the samples with and without the two proteins. Consequently, samples without proteins showed interconnected and open pores with more irregular shape. In the case of monomer ratio HEMA97%-AMPSA3% (without proteins), the morphological images revealed the presence of MMT clay within hydrogel surface (Figure 6a,b).

Samples with proteins (both HEMA97%-AMPSA3% and HEMA95%-AMPSA5%) revealed no MMT clay particles on the surface (Figure 6c,d). This fact may suggest that the modified clay is better distributed within the hydrogel matrix in the protein presence. Furthermore, they showed well defined and uniform pores with a narrow size. Finally, the proteins addition in the hydrogel represents a clear advantage for the hydrogel morphology in term of pore size distribution or pore shape.

#### 3.1.4. Swelling Behavior and Rheological Properties

**Figure 7:**
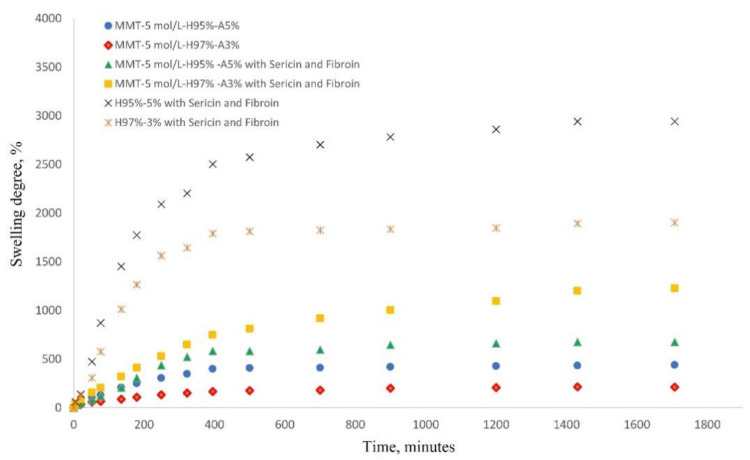
Swelling behavior of nanocomposite hydrogels in saline solution at 37 °C (see the legend).

The most important characteristic of a hydrogel is its ability to absorb and hold an amount of solvent in its network structure. The equilibrium swelling of a hydrogel material is a result of the balance of osmotic forces determined by the affinity to the solvent and network elasticity. Figure 7 shows the water swelling behavior of the nanocomposite hydrogels as follows: MMT-5 mol/L-H97%-A3% with Sericin and Fibroin, MMT-5 mol/L-H95%-A5% with Sericin and Fibroin, MMT-5 mol/L-H97%-A3%, MMT-5 mol/L-H95%-A5%, and the corresponding samples without modified MMT (control samples). As can be seen in Figure 7, the water swelling occurred rapidly, reaching equilibrium of water uptake in about 29 h. The hydrogel samples without modified montmorillonite showed an expected specific behavior of swelling degree increase due to the presence of hydrophilic monomer AMPSA and silk fibroin and sericin proteins. The swelling degree reached the value of 1900% for H97%-A3% with sericin and fibroin and 2941% for H95%-A5% with sericin and fibroin. When the modified MMT was present in the nanocomposite samples, it acted as a crosslinking agent resulting in a denser and more stable networking structure. This led to the decrease in the swelling degree of the hydrogels up to 1200% for MMT-5 mol/L-H97%-A3% with Sericin and Fibroin, and 675% for MMT-5 mol/L-H95%-A5% with Sericin and Fibroin.

**Figure 8:**
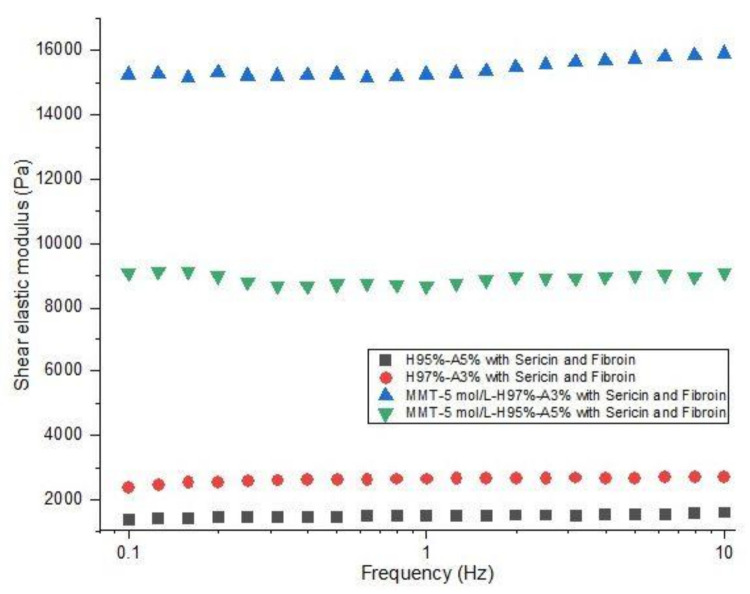
Rheological behavior of nanocomposite hydrogels: shear elastic modulus vs. frequency (see the legend).

The investigation of rheological properties was performed on swollen samples at swelling equilibrium. The stress was optimized to maintain a linear viscoelastic domain with frequency dependence. The dependence of elastic modulus (G′) on frequency (f) for the hydrogels is shown in Figure 8. The values of the elastic modulus of nanocomposite hydrogels with modified clay are 15200 Pa for MMT-5 mol/L-H95%-A5% with Sericin and Fibroin and 8000 Pa for MMT-5 mol/L-H97%-A3% with Sericin and Fibroin content. These values of G′ are far higher than the elastic modulus of samples containing no modified MMT (1500 and 2600 Pa). This elastic behavior is specific for crosslinked structures showing the importance of the modified clay as a crosslinking agent in this nanocomposite hydrogels. Only a physical distribution of the clay within the polymeric matrix could not have led to such good values for the rheological properties. The modified clay played the role of crosslinking sites in the hydrogel, probably resulting in a denser and more stable networking structure, which increases storage modulus of the nanocomposite hydrogels.

### 3.2. In Vitro Tests

#### 3.2.1. Biocompatibility Assessment of the Materials

**Figure 9:**
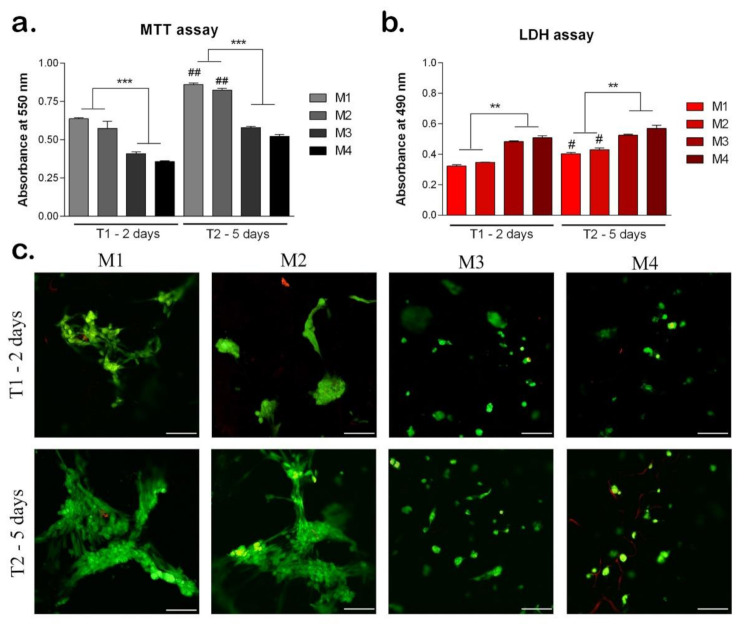
Biocompatibility of the materials seeded with hASC at T1 (2 days) and T2 (5 days). (**a**) Cell viability and proliferation rate evaluated by MTT test. ## *p* < 0.01; *** *p* < 0.001; ##-M1/M2 at T1 vs. T2. (**b**) Cytotoxicity levels induced by the materials evaluated by LDH test. # *p* < 0.05; ** *p* < 0.01; #-M1/M2 at T1 vs. T2. (**c**) Qualitative LiveDead analysis showing live cells (green) and nuclei of dead cells (red) of hASCs in materials. Scale bar 100 μm.

MTT assay indicated the cell viability and proliferation rate on the materials after 2 and 5 days of cell culture on the materials (Figure 9a). On all materials, cells proliferated between T1 and T2, indicating an increased cell viability. However, the levels of cell viability were statistically significant higher on sericin, and fibroin enriched materials (M1, M2) compared to M3 and M4, both at T1 and T2 (*p* < 0.001). This suggests the positive role of sericin and fibroin from the materials composition, on cells behavior. At T2, on M1 and M2, the cell viability levels were statistically significantly higher compared to T1 (*p* < 0.01), indicating cell proliferation, and suggesting the positive effect of these materials. Although there was no statistically significant difference between cell viability levels on M1 and M2, the highest level was observed on M1, both at T1 and T2. There is the same observation for M3 and M4 materials, with a higher level of cell viability on M3 material with 95% HEMA-AMPSA 5% compared to M4 material with 97% HEMA-AMPSA3%, suggesting the first composition could represent a better scaffold for cells.

The LDH test results confirmed the MTT test and indicated the cytotoxicity levels induced by the materials on cells (Figure 9b). At T1, the lowest levels of cytotoxicity were induced by M1 and M2 with similar values. Materials M3 and M4 induced statistically significant higher levels of cytotoxicity (*p* < 0.01) compared to M1 and M2, suggesting the enrichment with sericin and fibroin of M1 and M2 materials induced a better cellular response. At T2, the M1 and M2 materials induced the lowest levels of cytotoxicity suggesting the positive influence of their compositions on cellular behavior. At T2, M3 and M4 induced statistically significant higher levels of cytotoxicity compared to M1 and M2 (*p* < 0.01), the same as observed at T1.

The Live/Dead assay showed the live cells (green) and the nuclei of the dead cells (red) on all materials tested after 2 and 5 days of cell culture (Figure 9c). At T1, M1 and M2 materials promoted the highest level of cell viability. The cells were elongated, especially on M1 material suggesting the positive influence of the material composition on cellular behavior. Moreover, the cells clustered together which promoted their proliferation on M1 and M2 materials. On materials M3 and M4 there were lower levels of live cells compared to M1 and M2, which confirmed the MTT results. In addition, the cells retained a round shape, which indicates a poorer cellular response on these materials. At T2, cells proliferated on all materials, but at different rates. The highest number of live cells was observed on M1, with elongated aggregated cells. However, on M2, there was a similar level of live cells with the same behavior, suggesting the positive effect of sericin and fibroin from their composition [26,36,37]. Cells proliferated at a slower rate on M3 and M4 materials, and they mostly remained in the round shape, except on M3, where few cells started to elongate. This could indicate the better composition of 95% HEMA-AMPSA 5% (M3) compared to 97% HEMA-AMPSA 3% (M4), as the elongated form of the cells proves better adhesion. Cells may prefer the lower concentration of HEMA and higher of AMPSA (95%H–5%A) of M3 material, compared to the other concentrations in M4 material (97% HEMA-AMPSA 3%).

#### 3.2.2. Adipogenic Differentiation

**Figure 10:**
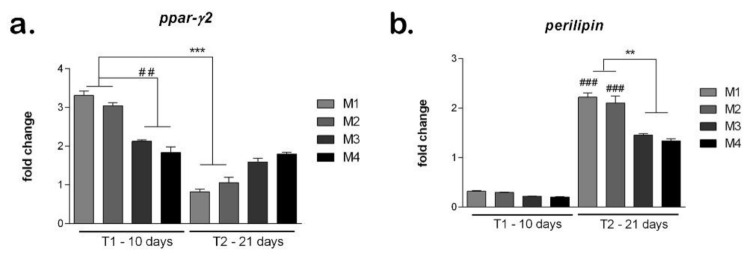
Evaluation of hASCs adipogenic differentiation on materials: (**a**) *Ppar-γ* expression after 10 and 21 days in differentiated cells on M1-M4 materials, obtained by qPCR, *** *p* < 0.001, ## *p* < 0.01; (**b**) *Perilipin* expression after 10 and 21 days in differentiated cells on M1-M4 materials, obtained by qPCR, ** *p* < 0.01, ### *p* < 0.001.

The development of adipogenic differentiation on materials was evaluated at gene level of adipogenic markers *ppar-γ2* and *perilipin* (Figure 10). The marker *ppar-γ2* expresses early in the development of adipogenesis and its expression is reduced later on the course of adipogenic differentiation. After 10 days of differentiation, the *ppar-γ2* levels were high on all materials tested, suggesting the process was induced [38]. There are statistically significantly (*p* < 0.01) higher levels of *ppar-γ2* in M1 and M2 compared to M3 and M4, indicating a more advanced status of differentiation induction. After 21 days, *ppar-γ2* levels are significantly reduced in M1 and M2 compared to T1, suggesting that adipogenesis was already induced. In M3 and M4, *ppar-γ2* levels are still high, suggesting that the differentiation process is in an early stage.

The *perilipin* levels are low on all materials tested, confirming the fact that this is a late marker that activates later during the differentiation process [39]. However, the *perilipin* levels are higher in M1 and M2 compared to M3 and M4, suggesting differentiation may be induced earlier on the first two materials. After 21 days, on all materials, the *perilipin* levels are high, suggesting that adipogenesis was induced and progressed. The highest *perilipin* level was on M1, which could indicate this material to be best suited to promote adipogenesis. Both in M1 and M2, perilipin was statistically significant (*p* < 0.001) higher at T2 compared to T1. M3 and M4 showed similar levels of *perilipin* that were statistically significant (*p* < 0.01) lower than M1 and M2, suggesting their composition (M1 and M2) could be better for soft tissue engineering applications.

### 3.3. In Vivo Biocompatibility and Inflammatory Response

**Figure 11:**
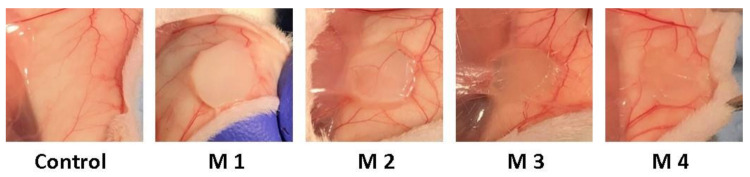
Photographs taken 3 weeks post-implantation (control group-without any mate-rial; M1 group-material containing MMT-5 mol/L-H95%-A5% with Sericin and Fibroin; M2 group-material containing MMT-5 mol/L-H97%-A3% with Sericin and Fibroin; M3 group-material containing MMT-5 mol/L-H95%-A5%; M4 group-material containing MMT-5 mol/L-H97%-A3%).

After 3 weeks post-implantation, there were no inflammatory, edematous, exuded, or adherent areas in the case of any implanted material observed. Macroscopically the implanted materials seem to be well tolerated, a well-developed vascular network being established around them. During the 3-week experimental period, the animals did not show inflammatory reactions at the implant level, except the normal postoperative ones and the skin above the implant did not show pathological macroscopic lesions (Figure 11).

**Figure 12:**
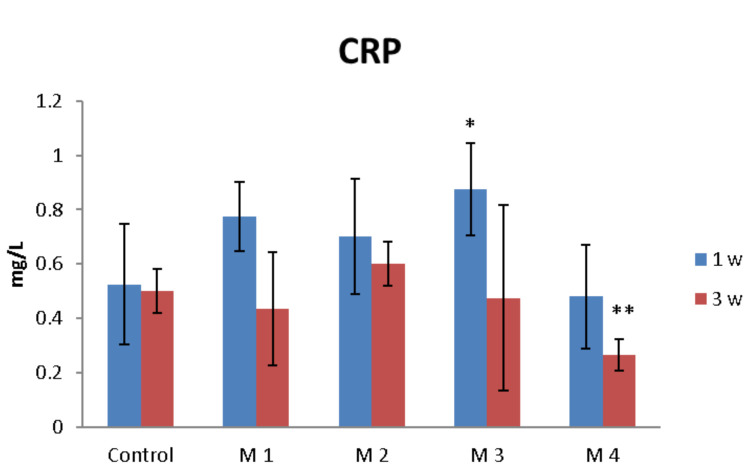
The effects of nanocomposite hydrogels subcutaneous implantation in mice on the C-reactive protein (CRP) levels at 1- and 3-weeks post-surgery. * *p* < 0.05, ** *p* < 0.01 (*/** comparison between M3/M4 and control).

C-reactive protein (CRP) is a proinflammatory “trigger” because it stimulates the monocyte production of IL-1, IL-6 and TNF-α. Figure 12 shows the effects of nanocomposite hydrogels implanted subcutaneously in mice on the CRP serum level.

**Figure 13:**
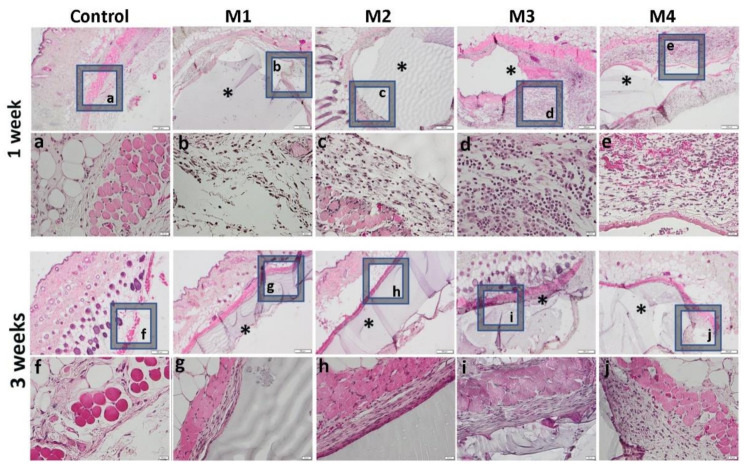
Representative histological images of subcutaneous explants with HEMA/AMPSA hydrogels supplemented with fibroin/sericin, at 1 and 3 weeks after implantation. (control group without any material; M1 group-material containing MMT-5 mol/L-H95%-A5% with Sericin and Fibroin; M2 group-material containing MMT-5 mol/L-H97%-A3% with Sericin and Fibroin; M3 group-material containing MMT-5 mol/L-H95%-A5%; M4 group-material containing MMT-5 mol/L-H97%-A3%; * material; HE staining, ob.4×, 20×; a-j 20× magnifications of 4× pictures).

**Figure 14:**
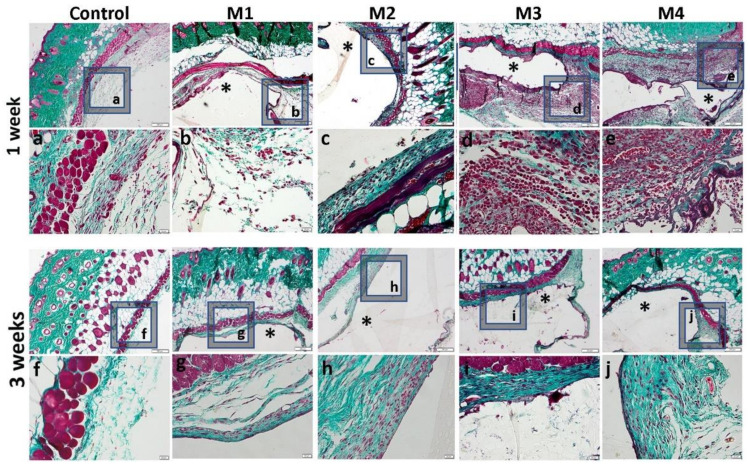
Histological analysis of the biocompatibility of HEMA/AMPSA hydrogels supplemented with fibroin/sericin, at 1 and 3 weeks after subcutaneous implantation (control group-without any material; M1 group-material containing MMT-5 mol/L-H95%-A5% with Sericin and Fibroin; M2 group-material containing MMT-5 mol/L-H97%-A3% with Sericin and Fibroin; M3 group-material containing MMT-5 mol/L-H95%-A5%; M4 group-material containing MMT-5 mol/L-H97%-A3%; * material; Gomori Trichrome staining, ob.4×, 20×; a-j 20× magnifications of 4× pictures).

One week after the subcutaneous implantation of the materials, extravasation of PMN in the peri-implant connective tissue is noticed, the acute inflammatory reaction being higher for the basic materials of Material 3 with 95% HEMA and 5% MMT and Material 4 with 97% HEMA and 3% MMT, and lower for Material 1 with 95% HEMA and 5% MMT + sericin and fibroin and Material 2 with 97% HEMA and 3% MMT + sericin and fibroin. At 3 weeks post-implantation, lymphocytes and macrophages are observed, especially attached to the surface of the material and rare multinucleated giant cells, which suggests a moderate foreign body reaction, especially for materials coated with sericin and fibroin (Figure 13). This observation is also reinforced by the analysis of the proliferation of collagen in trichrome staining, the fibrous capsule around the implant at 3 weeks timepoint being lower for Material 1 with 95% HEMA and 5% MMT + sericin and fibroin and Material 2 with 97% HEMA and 3% MMT + sericin and fibroin, compared to materials 3 and 4 (Figure 14).

**Figure 15:**
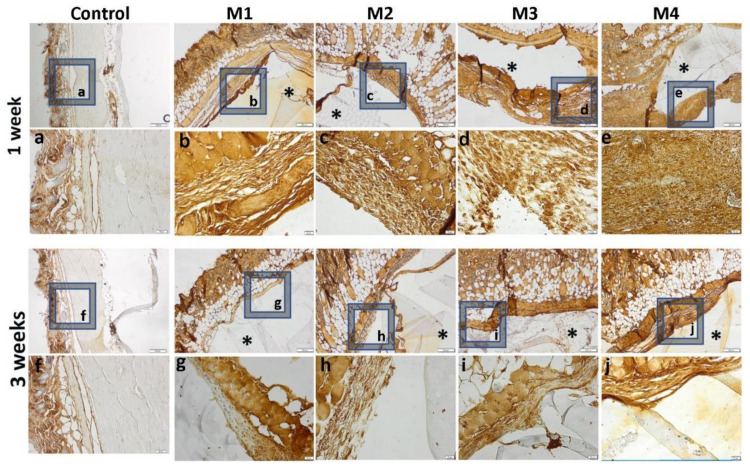
Immunohistochemical expression of TNFα and tissue-specific distribution at 1 week and 3 weeks post- implantation (control group-without any material; M1 group-material containing MMT-5 mol/L-H95%-A5% with Sericin and Fibroin; M2 group-material containing MMT-5 mol/L-H97%-A3% with Sericin and Fibroin; M3 group-material containing MMT-5 mol/L-H95%-A5%; M4 group-material containing MMT-5 mol/L-H97%-A3%; * material; ob.4×, 20×; a-j 20× magnifications of 4× pictures).

The inflammatory reaction is more intense in the case of materials 3 and 4 and de-creases when coated with sericin and fibroin, highlighted by a lower immunopositivity of TNFα at the peri-implant level (Figure 15).

## 4. Discussion

Tissue engineering and regenerative medicine strive to find appropriate substitutes for damaged organs or tissues and promote their regeneration. Choosing suitable substitutes for soft tissue defects depends greatly on the qualities of the material chosen and the abilities of the cells to adhere, proliferate and differentiate.

The developed nanocomposite hydrogels are special polymeric-based materials, which combine the advantages of both hydrogel- and nanocomposite-like materials. Such materials present a superior network design with respect to conventional hydrogels which are crosslinked based on low molecular weight crosslinking agents. The idea of using modified inorganic nanoparticles as crosslinking agents leads to special materials. They show extraordinarily stretchability, flexibility, and high fracture toughness [40,41,42]. The tested nanocomposite hydrogels were designed and developed based on our previous expertise. Therefore, our previous work showed nanocomposite materials with similar design. The nanocomposite materials were based on modified magnetic nanoparticles with double reinforcing and crosslinking role. The results revealed that nanocomposites presented a special morphological design with a superior distribution of inorganic nanoparticles within polymeric matrix. The dynamic mechanical investigation showed a good elastic behavior in stress conditions [43]. Furthermore, a similar result was obtained in the case of modified layered clays. Morphological and structural investigation showed good predictable results with a high compatibility and mechanical resistance [44].

The developed nanocomposite hydrogels proved to be biocompatible and induce low cytotoxicity. Each component of the newly developed nanocomposite hydrogels was previously shown to have a positive impact on the cells behavior when put in contact with them. A number of studies investigated the potential of different combination of materials for hydrogels used for soft tissue applications. In a study by Kim et al. [5], a hydrogel based on 2-hydroxylethylmethacrylate (HEMA) and acrylamide (Am) copolymer (Poly(HEMA-Am)) was under investigation for potential use in breast reconstruction. Human fibroblasts (hFBs) and human adipose-derived stem cells (hADSCs) were put in contact with poly (HEMA-Am) and cytotoxicity was tested using EZ-cytox and Live/Dead assays. Their results showed that cell survival rate was approximately 80%, which indicated a good biocompatibility of the HEMA hydrogel. Moreover, in another study, poly(2-hydroxyethyl methacrylate-co-2-acrylamido-2-methylpropane sulfonic acid) (p(HEMA-AMPSA)) hydrogel was developed. Fibroblasts were cultured in the hydrogels and cytotoxicity and cell viability was assessed. Not only was the cytotoxicity of the hydrogel low, but it also promoted spindle-shaped morphology of the cells, indicating good cellular adhesion and biocompatibility [7].

Besides mechanical and structural properties, polymeric nanocomposites based on layered clays revealed enhanced biocompatibility due to clay presence [45]. The silk proteins (sericin and fibroin) clearly influenced the biocompatibility effect toward developed M1 and M2. The coating of the basic materials (3 and 4) with sericin and fibroin determined a lower foreign body reaction (inflammatory reaction, peri-implant fibrous capsule), being therefore more biocompatible. This behavior was also characteristic for our previously silk fibroin containing hydrogels [18]. In another study [46] silk fibroin was physically deposited onto the surface of a Ti6Al4V biometal to ensure a strong interface between the protein and the metallic implant.

Furthermore, an interesting approach to promote the osteointegration of titanium metallic implants is based on the use of bioactive molecules. In this respect, silk sericin was immobilized onto the surface of titanium using glutaraldehyde as crosslinking agent [47]. In vitro assessment of osteoblast cells cultured onto the surface of metallic and sericin-immobilized metallic surfaces showed more viable cells with sericin coating. One may notice increased adhesion, proliferation, and differentiation of osteoblast cells [47]. Silicone implants were coated with spider silk fibroin in a study by Zeplin et al. [48], and those materials showed increased biocompatibility in the presence of the spider protein. Decreased foreign body reaction and a reduced capsule behavior were seen, therefore protein coating could be a successful solution to increase the biocompatibility of various medical implants [48,49].

The same silk fibroin proved to be effective as a component in flexible electronic coatings for implantable and wearable devices [50]. The presence of silk protein coatings was revolutionary for electronic devices having great premises for future use in computing technology, human-machine interfaces and medical diagnosis [51].

Another important aspect considered for tissue engineering is represented by the cells used in the study. According to the future application, certain cells could be more appropriate. For our study, we chose to use stem cell isolated from adipose tissue, taking into consideration all the advantages their use brings forward. They are easily obtainable and can differentiate toward many lineages. Moreover, being isolated from adipose tissue, they possess certain characteristics specific to soft tissues. In our study, hASCs were seeded in the nanocomposite hydrogels to evaluate the material’s biocompatibility and its potential to sustain cells’ adipogenic differentiation. Hydrogels proved to be biocompatible, especially the ones enriched with sericin and fibroin, with more live elongated clustered cells which promoted their proliferation, adhesion and survival. Furthermore, hASCs were induced to differentiate toward adipogenic lineage after being seeded in hydrogels. A key regulator of adipogenesis is peroxisome proliferating activated receptor gamma-2 (PPARγ2), which is expressed in the early stages of differentiation [38]. Another marker of adipogenic differentiation is considered perilipin, a protein that coats the lipid droplets formed in the late stages of differentiation [39].

In the present study, the materials provided a good substrate for the adipogenic differentiation of hASCs, as gene expression level of *ppar-γ2* was higher after 10 days of differentiation compared to 21 days. In addition, *perilipin* gene expression levels were lower at the beginning of differentiation induction compared to the expression levels after 21 days. Such results are confirmed by other studies as well which evaluated the expression levels of *ppar-γ2* and *perilipin*, as indicators of adipogenesis [52,53]. Addition of sericin and fibroin in the M1 and M2 materials greatly enhanced their effect on adipogenic differentiation of the cells. In a study by Dinescu et al. [20], addition of sericin in a collagen scaffold stimulated the overexpression of PPARγ2 in hASCs and also the upregulated transcription of FAS, aP2 and perilipin markers. As such, it was concluded the efficiency of adipogenesis to be correlated with the presence of sericin in the material’s composition.

To further validate the newly developed nanocomposite hydrogels for future use in soft tissue engineering, the materials were implanted into a subcutaneous pocket in mice and their biocompatibility was evaluated. The inflammatory reaction was more intense for materials without sericin and fibroin (M3 and M4) than compared to the reaction to materials with sericin and fibroin (M1 and M2). This was also highlighted by a lower immunopositivity of TNFα at the peri-implant level. Other studies confirmed the positive impact of sericin and fibroin addition in materials in in vivo studies. Sericin hydrogels were shown to reduce inflammation and promote wound healing in mice [54]. Fibroin-impregnated polyester grafts were implanted to in vivo models and showed less foreign body and inflammation reactions compared to grafts without fibroin, proving its positive role [55].

Considering the premises of a nanocomposite hydrogel design tailored with high biocompatible silk proteins, the developed M1 and M2 materials, enriched with sericin and fibroin, are expected to highlight suitable features in regenerative medicine for soft tissue uses.

## Figures and Tables

**Table 1:** Recipes for hydrogel preparation.

Materials	Monomer Concentration (mol/L)	HEMA:AMPSA (%)	KPS * Molar	MMT (*w*/*v* %)	Sericin (*w*/*v* %)	2% Fibroin Solution (*v*/*v* %)
M1	5	95:5	1%	1%	0.5%	15%
M2	5	97:3	1%	1%	0.5%	15%
M3	5	95:5	1%	1%	-	-
M4	5	97:3	1%	1%	-	-

* with respect to monomer number of moles.

## Data Availability

Not applicable.
